# Supplementary material for: Two centuries of forest succession, and 30 years of vegetation changes in permanent plots in an inland sand dune area, The Netherlands
Source: PLoS One. 2021 Apr 29;16(4):e0250003. doi: 10.1371/journal.pone.0250003 (PMC8084203; doi:10.1371/journal.pone.0250003)
Supplement: S1 Table — Species are sorted according to their participation in the succession. Frequencies and covers of woody species are combined in a single layer. (DOCX) [file pone.0250003.s001.docx]

**S1 Table.** Species frequencies (Fr) and average % covers (Co) in plots A, B and C in the years of observation. Species are sorted according to their participation in the succession. Frequencies and covers of woody species are combined in a single layer.

Plot and sampling year A 1988 B 1988 C 1988 C 2018

Number of plots 400 400 400 400

Age range 0–40 30–112 110–175 140–205

Fr Co Fr Co Fr Co Fr Co

**Common species:**

*Festuca arenaria*  16 1.4 . . . . . .

*Ammophila arenaria*  27 0.8 1 0.0 . . . .

*Corynephorus canescens*  64 3.4 22 1.4 . . . .

*Festuca ovina*  13 0.4 58 5.4 3 0.1 . .

*Agrostis vinealis*  18 1.1 29 1.3 1 0.0 1 0.0

*Spergula morisonii*  10 0.2 22 0.7 1 0.0 . .

*Polytrichum piliferum*  14 2.0 23 3.0 1 0.0 . .

*Carex arenaria*  1 0.0 31 1.7 . . . .

*Pinus sylvestris*  29 6.4 100 35.5 100 35.4 100 45.4

*Deschampsia flexuosa*  7 0.2 96 47.0 100 35.9 100 22.9

*Prunus serotina*  8 0.3 57 1.2 17 0.4 58 1.1

*Sorbus aucuparia*  4 0.1 64 1.3 26 0.4 74 1.1

*Amelanchier lamarckii*  1 0.0 21 0.2 5 0.1 32 0.4

*Ceratocapnos claviculata*  2 0.1 20 0.4 2 0.0 19 0.4

*Rhamnus frangula*  1 0.0 63 1.8 79 2.6 95 3.0

*Quercus robur*  3 0.0 32 0.7 68 1.7 75 1.9

*Empetrum nigrum*  3 0.1 25 2.4 69 14.8 80 9.9

*Betula pendula + B. pubescens*  10 0.7 47 4.2 27 0.6 85 4.0

*Vaccinium myrtillus*  1 0.0 2 0.0 19 0.4 97 9.9

*Fagus sylvatica*  1 0.0 2 0.0 11 0.9 63 3.6

*Lonicera periclymenum*  . . 3 0.1 2 0.0 11 0.2

*Molinia caerulea*  . . 7 0.2 1 0.0 10 0.2

*Pseudotsuga menziesii*  . . 1 0.0 2 0.0 14 0.3

*Galium saxatile*  . . 1 0.0 4 0.1 18 0.5

*Dryopteris carthusiana* agg. 1 0.0 2 0.0 4 0.0 30 0.6

*Vaccinium vitis-idaea*  . . . . 4 0.1 44 4.8

*Ilex aquifolium*  . . . . 1 0.0 51 0.8

**Rare species:**

*Juncus squarrosus*  2 0.0 . . . . . .

*Cerastium fontanum*  1 0.0 . . . . . .

*Jasione montana*  1 0.0 . . . . . .

*Poa annua*  1 0.0 . . . . . .

*Senecio vulgaris*  1 0.0 . . . . . .

*Hypochaeris radicata*  1 0.0 1 0.0 . . . .

*Taraxacum* spp. 1 0.0 1 0.0 . . . .

*Senecio sylvaticus*  2 0.0 1 0.0 1 0.0 . .

*Erica tetralix*  1 0.0 . . 1 0.0 . .

*Dryopteris filix-mas*  . . 1 0.0 . . . .

*Malva sylvestris*  . . 1 0.0 . . . .

*Polygonatum multiflorum*  . . 1 0.0 . . . .

*Polypodium vulgare*  . . 1 0.0 . . . .

*Quercus petraea*  . . 1 0.0 . . . .

*Chamerion angustifolium*  . . 2 0.0 1 0.0 . .

*Calluna vulgaris*  7 0.4 3 0.0 6 0.1 1 0.0

*Rumex acetosella*  2 0.0 1 0.0 . . 3 0.1

*Holcus lanatus*  1 0.0 . . . . 1 0.0

*Aira praecox*  . . . . 1 0.0 . .

*Dryopteris* spp. . . . . 1 0.0 . .

*Carex pilulifera*  . . . . 1 0.0 1 0.0

*Juniperus communis*  . . . . 1 0.0 1 0.1

*Picea abies*  . . . . 1 0.0 1 0.0

*Rubus* spp. . . . . . . 6 0.1

*Digitalis purpurea*  . . . . . . 4 0.1

*Quercus rubra*  . . . . . . 3 0.0

*Agrostis capillaris*  . . . . . . 3 0.1

*Acer pseudoplatanus*  . . . . . . 1 0.0

*Castanea sativa*  . . . . . . 1 0.0

*Larix* spp. . . . . . . 1 0.0

*Rumex acetosa*  . . . . . . 1 0.0

*Sorbus aucuparia* . . . . . . 1 0.0
