# Supplementary material for: Two centuries of forest succession, and 30 years of vegetation changes in permanent plots in an inland sand dune area, The Netherlands
Source: PLoS One. 2021 Apr 29;16(4):e0250003. doi: 10.1371/journal.pone.0250003 (PMC8084203; doi:10.1371/journal.pone.0250003)
Supplement: S2 Table — All considered variables explained 23.0% of total variation (year of sampling; successional age; P–Plain; PD–Plateau dunes; LD–Low dunes; HD–High dunes). Interactions of two variables are marked with an asterisk. P values were adjusted by the Holm correction. (DOCX) [file pone.0250003.s002.docx]

**S2 Table.** Marginal effects of environmental variables in partial RDA analysis. All considered variables explained 23.0% of total variation (year of sampling; successional age; P – Plain; PD – Plateau dunes; LD – Low dunes; HD – High dunes). Interactions of two variables are marked with an asterisk. P values were adjusted by the Holm correction.

Variable Explains % pseudo-F P P(adj)

Year 13.7 191 0.001 0.010

Age 7.5 96.6 0.003 0.024

Age*P 4.9 62.3 0.005 0.035

P 4.6 57.6 0.006 0.036

PD 2.9 36.4 0.002 0.018

Age*PD 2.9 36.3 0.006 0.036

Age*LD 1.2 14.8 0.065 0.260

LD 1.0 11.6 0.124 0.366

HD 0.9 10.9 0.122 0.366

Age*HD 0.9 10.3 0.138 0.366
